# Life-history and genetic relationships in cooperatively breeding dwarf mongoose groups

**DOI:** 10.1098/rsos.241125

**Published:** 2024-10-02

**Authors:** Josh J. Arbon, Amy Morris-Drake, Julie M. Kern, Gabrielle M. K. Howell, Jeanette Wentzel, Andrew N. Radford, Hazel J. Nichols

**Affiliations:** ^1^ School of Biological Sciences, University of Bristol, Bristol BS8 1TQ, UK; ^2^ School of Environmental and Rural Science, University of New England, Armidale, New South Wales 2351, Australia; ^3^ Department of Biosciences, University of Swansea, Swansea SA2 8PP, UK; ^4^ Department of Wildlife Studies, Faculty of Veterinary of Science, University of Pretoria, Onderstepoort, Pretoria, Gauteng, South Africa; ^5^ Hans Hoheisen Wildlife Research Station, Department of Veterinary Tropical Diseases, University of Pretoria, Onderstepoort, Pretoria, Gauteng, South Africa

**Keywords:** cooperative breeding, social evolution, dispersal, genetic relatedness, helping behaviour, reproductive skew

## Abstract

Cooperatively breeding societies show distinct interspecific variations in social and genetic organization. Long-term studies provide invaluable data to further our understanding of the evolution and maintenance of cooperative breeding but have also demonstrated how variation exists within species. Here we integrate life-history, behavioural and genetic data from a long-term study of dwarf mongooses *Helogale parvula* in South Africa to document mating, breeding, dispersal and relatedness patterns in this population and compare them to those found in a Tanzanian population at the other extreme of the species’ range. Our genetic data reveal high levels of reproductive skew, above that expected through observational data. Dispersal was male-biased and was seen more frequently towards the onset of the breeding season, but females also regularly switched between groups. These patterns of breeding and dispersal resulted in a genetically structured population: individuals were more related to groupmates than outsiders, apart from the unrelated dominant pair, ultimately resulting in reduced inbreeding risk. Our results also demonstrate that dwarf mongooses are largely consistent in their social structure across their sub-Saharan distribution. This work demonstrates the direct and indirect pathways to reproductive success for dwarf mongooses and helps to explain the maintenance of cooperative breeding in the species.

## 1. Introduction

Cooperatively breeding societies are those in which reproducing individuals are assisted in the rearing of their offspring by non-breeding subordinate helpers. Cooperative breeding has been widely reported in mammals, birds and invertebrates, as well as some fish, and is apparent in a variety of environments [1–4]. It is therefore unsurprising that there are large interspecific differences in key facets of this social system. For instance, while cooperative-breeding groups in many species comprise a single reproductive pair and a number of subordinate helpers [5–7], a diversity of other social structures is also observed, such as those where many females breed (as in the banded mongoose *Mungos mungo* [8]) or multiple pairs combine breeding efforts (as in the greater ani *Crotophaga major* [9]). Some species (e.g. meerkats *Suricata suricatta*, dwarf mongooses *Helogale parvula* and chestnut-crowned babblers *Pomatostomus ruficeps*) are obligate cooperative breeders—breeding pairs are unable to reproduce successfully without help from non-breeders [10–13]—while in others (facultative cooperative breeders such as yellow-bellied marmots *Marmota flaviventris* and black-throated tits *Aegithalos concinnus*), breeding can be successful with or without help depending on conditions [14–16]. The number of helpers per group can vary from one to many; in extreme insect cases, there can be thousands of non-breeding workers [17]. Helpers are often delayed dispersers [18], as seen in Seychelles warblers *Acrocephalus sechellensis* and African wild dogs *Lycaon pictus*, such that the sex of helpers is linked to dispersal strategies [11,19,20]. Helpers can also be unrelated to the breeding pair; in many cooperatively breeding systems, including meerkats and carrion crows *Corvus corone*, some helpers are immigrants born outside the natal group and who therefore help to raise unrelated young [21–23]. Such variation between taxa therefore results in a wide range of patterns of relatedness between different cooperatively breeding systems, with impacts on social organization and behaviour.

Our understanding of cooperative breeding has been greatly enhanced by long-term studies of uniquely recognizable individuals [24]. For example, populations of meerkats, banded mongooses, long-tailed tits *Aegithalos caudatus*, pied babblers *Turdoides bicolor* and superb starlings *Lamprotornis superbus* have been monitored closely for decades, with uniquely marked individuals followed throughout their lifetimes and across multiple generations [25–29]. The resulting wealth of behavioural and life-history observations has enabled insight into factors underlying which individuals help, when they provide assistance and how much they contribute to cooperative actions [15,21,30–32]. However, without genetic information, we cannot fully understand the intricacies of individual relationships and group dynamics. The inclusion of genetic analyses to long-term studies often reveals patterns of breeding and relatedness different from those that would be expected through behavioural observation alone [33–36]. Helping behaviour is often correlated with helper relatedness, such as in noisy miners *Manorina melanocephala*, but relatedness is not always accurately assessable through field observations [37]. A striking disconnect between relatedness and observations comes from superb fairy-wrens *Malurus cyaneus* where, despite a lack of observed extra-pair copulations, genotyping revealed 95% of broods contained extra-pair young [38]. Similarly, genetic analysis found a high prevalence of out-group paternity in banded mongooses [39], highlighting a key conflict of interest between females who seek out-group matings at the expense of costly conflict among males [40].

Many cooperative breeders are found across a wide geographic range, so within-species variation is expected [4,41,42]. For example, the prevalence of helping can differ substantially between populations. In an extreme case, carrion crows are monogamous in the majority of their European range but facultative cooperative breeders in a region of northern Spain [43]; interpopulation cross-fostering has demonstrated that this behaviour is not genetically driven [44]. In species where cooperative breeding is seen in different populations, the level of alloparental care can differ. For instance, in Arctic foxes *Alopex lagopus*, the percentage of breeding events where there is helper care ranges from *ca* 5% in Canada to *ca* 50% in Russia and more than 80% in Sweden [45–47]. Variation in within-group relatedness also occurs: in one population of pukeko *Porphyrio porphyrio*, characterized by habitat saturation, groups of close relatives bred and maintained a territory year-round, while in a different habitat at lower saturation, non-breeding flocks formed in the winter and groups of unrelated individuals formed in the breeding season to raise young [48]. In green woodhoopoes *Phoeniculus purpureus*, there are various differences between populations in the extremes of their range: for example, groups in Kenya can be as large as 16 and breed up to three times per season, with over 90% of breeders assisted by helpers; by contrast, groups in South Africa have a maximum group size of eight and only breed once per year, with over 30% of pairs breeding without help [49,50]. Documenting similarities and differences between populations in terms of their behaviour, relatedness and social organization, as well as considering what ecological variation might underpin differences, is therefore important in helping us understand the evolution of cooperative traits [43].

Dwarf mongooses are a small group-living carnivore found across a wide geographic range, throughout eastern and southern Africa [51]. The Dwarf Mongoose Research Project (DMRP) was set up in 2011 to study the behaviour of wild groups in South Africa, amassing 12 years of life-history and behavioural data. In addition, non-invasive faecal sampling allowed genotyping of the population in 1 year. A dwarf mongoose population from Tanzania was similarly well studied between 1974 and 1987: in this population, dwarf mongooses live in groups with a mean of nine members, comprising a dominant breeding pair and subordinate helpers, with no difference in weight between sexes [52]. Groups in Tanzania produce up to three litters of pups during the rainy wet season (November–June [52]); a dominant pair partially suppress subordinate reproduction, resulting in a reproductive skew of around 80% [33,53–55]. Movement between groups occurs in both sexes, but is male-skewed, with more females remaining in their natal group [56,57]. Group movements do not, however, prevent inbreeding as relatedness between dominant breeding pairs is similar to that of other potential opposite-sex groupmates [57]. In this paper, we integrate our long-term behavioural and life-history observations with genetic data to describe the social structure and relatedness of cooperatively breeding dwarf mongooses in South Africa. We use updated genetic methods to offer new insights, with the overall goal of furthering our understanding of dwarf mongoose sociality and the evolution of cooperative breeding more generally. We compare our findings with those from the population studied in Tanzania, at the opposite end of the species’ range.

## 2. Methods

### 2.1. General data collection

Our data were collected at the DMRP on Sorabi Rock Lodge, a 4 km^2^ private game reserve in Limpopo Province, South Africa (24.20 S, 30.78 E). The study site is part of the savanna biome characterized by warm, wet summers and cool, dry winters. Data were collected between 2012 and 2023 (the study period) from 13 groups of wild dwarf mongooses habituated to close observation (<5 m); not all groups were monitored in all years. We carried out genetic analysis on samples collected between May and July 2018 (the sampling period). This genetic sampling was originally planned to form the basis of a longer-term study into relatedness and cooperative behaviour, but due to challenges surrounding the COVID-19 pandemic and the subsequent cessation of the DMRP, this was not possible. Individuals were identifiable via unique visual characteristics such as scars or blonde dye marks (Wella UK Ltd, Surrey, UK) that were added to their fur by observers using an elongated paintbrush. Study individuals were trained to stand on a balance scale for a small reward of crumbed hard-boiled egg. All research was conducted under permission from the Limpopo Department of Economic Development, Environment and Tourism (permit number: 001-CPM403-00013), and ethical approval from the University of Pretoria, South Africa (Animal Ethics Committee: NAS321/2022) and the University of Bristol, UK (Animal Welfare and Ethics Review Body: UIN/17/074), and in line with the Association for the Study of Animal Behaviour (ASAB) guidelines for the ethical treatment of animals [58]. All faecal samples were collected under Section 20 permit from the Department of Agriculture, Forestry & Fisheries, Republic of South Africa (reference: 12/11/1/7/3), while extracted DNA was transported and exported with the appropriate permissions from the state veterinarian of Limpopo Province.

Groups were monitored by an observer from just after sunrise when the first individual emerged from the overnight sleeping burrow until the last individual entered the sleeping burrow at the end of the day, around sunset. During the day, groups were followed on foot while they moved through their territory, with key life-history information and behaviours recorded. Matings were recorded ad libitum, noting the identities of the male and female involved. Pregnancy was identified via observation of distention of the female abdomen and confirmed through consistent body-mass gain across the gestation period. As multiple females can become pregnant synchronously, pregnancy events were defined as times where one or more females in a group were noted as pregnant within a two week period. The birth or loss of a litter was identified by the change in appearance of the pregnant female(s), their body-mass loss and sudden behavioural changes in group members, such as the constant presence of a babysitter at the burrow [59]. Emergence was recorded as the date at which pups appeared above ground from the burrow unassisted. Body masses were recorded at the morning sleeping burrow before the group left to commence foraging. Here we present body-mass data for individuals a minimum of 1 year old after the major growth period has ended.

Group composition was recorded daily for each visited group. The composition of individuals was used to calculate group size on a given day. For the purposes of calculating group size, individuals of unknown origin moving between groups were assigned a conservative minimum age of six months, which is younger than any recorded group switch in the system. Individuals were considered to have switched groups when (i) an unknown individual appeared in a habituated group; (ii) a known individual moved between two habituated groups; or (iii) a known individual left a habituated study group and was later seen with an unhabituated group that was not in the study population. We also recorded information on whether individuals switched groups on their own or in a coalition. All individuals that disappeared from a habituated study group but were never seen in another group were not considered to have switched groups, as it is not possible to distinguish between mortality and group-switching in these cases.

### 2.2. Life-history statistical methods

All data were subject to a series of cleaning procedures and integrity checks both in the field on the collection and once added to the project database. We defined breeding seasons as starting at the onset of oestrus in the population (typically September) and running until the last litter in the population had emerged from the breeding burrow for the first time (typically March; as in Morris-Drake *et al*. [60]). The remainder of the year was classified as the non-breeding season. Breeding seasons therefore largely coincide with the hot, wet, summer months, and non-breeding seasons the cooler, dry, winter months. Group-seasons refer to one season in one group; i.e. the 2014−2015 breeding season in the BW group is one group-season. For body mass and subordinate number, we only analyse data from the breeding season to (i) increase independence of data year on year and (ii) enable direct relation to breeding and genetic measures. For data on group-switch events, we analyse data from the whole year as these are much rarer events and their occurrence, even if during the non-breeding season, has direct relevance to group compositions, reproductive opportunity and helping in the breeding season. We calculated summary metrics, for both mating/breeding and group composition, by taking the values for each group-season, generating a group-mean across seasons and then taking the mean of these group-means to prevent any one group introducing undue bias into the estimates. We inferred group sizes for days where the group was not observed as the mean of the group size observed immediately before and after the unobserved period (linear interpolation), which in the majority of cases is the same due to the stability of group composition. This resulted in a group size value for every day within the given period, which was then averaged within-group to produce a weighted group size.

We conducted all life history and behavioural data analysis in R version 4.4.0 [61]. We ran linear mixed models (LMMs) using the R package ‘lme4’ [62] to assess whether there were any sex differences in the number of subordinates per group or in adult body mass. Mean number of subordinates and mean adult body mass were calculated for each sex per group-season. To calculate mean body mass, we first took individual mean body mass across a group and season, then took the mean of these values for each sex (e.g. mean of all mean weights of X male adults in group Y in season Z). For models investigating body mass and number of subordinates, sex was fitted as a fixed factor and both group and season were fitted as random factors. To assess whether there was a sex bias in dispersal in coalitions, we ran a binomial generalized linear model with coalition (Y/N) as a binary response term and the sex of the dispersing individual as a fixed factor and group-switch event as a random factor. A one-sample *t* test was conducted to evaluate whether the change in number of same-sex competitors following an individual switching groups was different from zero. Values of *p* for linear models were derived from likelihood ratio tests (LRTs) between the full model and the full model minus the variable of interest. Model fit and assumptions were checked using the package DHARMa [63].

### 2.3. Genetics methods

#### 2.3.1. Sample collection

We collected faecal samples in the field immediately after they were deposited. Samples were only collected if visual ID on the individual defecating was ensured. We discounted faecal deposits for collection if they were immediately adjacent to, or in direct contact with, other faeces from different individuals to reduce the risk of cross-contamination. On collection, we placed samples immediately in ethanol and chilled them with ice packs while transported to the field station. Following the method proposed by Nsubuga *et al*. [64], we stored samples in ethanol for 24−48 h, at which point they were transferred to silica beads to ensure full dehydration. We stored samples frozen in this way until extraction.

#### 2.3.2. Microsatellite genotyping

We extracted DNA from faecal samples using Qiagen^®^ QIAmp Stool Kit DNA extraction kits (Qiagen, Venlo, The Netherlands), following the manufacturer’s instructions with modifications suggested in Wehausen *et al.* [65]. We then genotyped samples using a panel of 13 microsatellites (electronic supplementary material, table S1). Genotyping was conducted using multiplex PCRs (Qiagen^®^ Multiplex PCR Kit, UK) with fluorescent labelled forward primers following the manufacturer’s recommendations (but was conducted in 12 µl reactions to maximize cost-effectiveness). We visualized PCR products through fragment-size analysis on an ABI 3730 DNA analyser (Applied Biosystems, Waltham, MA, USA) and scored allele sizes using GeneMapper^®^ Software Version 4.0 (Applied Biosystems, Lincoln, USA). To maximize genotype quality, we manually inspected all traces, and genotype calls were corrected where necessary.

DNA from non-invasive sources often has lower amplification success or greater rates of allelic dropout than tissue or blood [66]. To obtain reliable genotypes, we initially genotyped three faecal samples per individual. The three genotypes were then combined into a consensus genotype, considering the evidence for allelic dropout and misleading stutter or spurious bands. This method produced unambiguous genotypes with good coverage in the majority of cases (*n* = 81 individuals). However, there were some gaps in the genotypes that required filling. We therefore re-genotyped individuals at a further two samples if (i) they failed to amplify at least 10 loci (10 individuals) or (ii) any mismatching genotypes were present that were not explained by occasional allelic dropout (eight individuals).

Our final dataset contained genotypes for 94 individual dwarf mongooses, accounting for 94% of individuals across seven social groups. The mean number of loci genotyped per individual was 11.9 (range 2−13), and 80% of individuals had genotype data at a minimum of 12 of the 13 microsatellite loci. We conducted tests for Hardy–Weinberg equilibrium (HWE) and calculations of null allele frequency and diversity indices using Cervus 3.0.7 [67]; outputs are displayed in electronic supplementary material, table S2. None of the microsatellites deviated from HWE, and all had estimated null allele frequencies below 0.05. Our microsatellites had a mean polymorphic information content of 0.57, a mean expected heterozygosity of 0.61 and a probability of identity of 7.86 × 10^–11^, indicating that the microsatellite panel was sufficiently diverse for our downstream analyses.

#### 2.3.3. Population genetics

We used *F*-statistics to assess the level of genetic structuring present within and between dwarf mongoose groups. *F*-statistics measure genetic differentiation among groups by comparing observed levels of heterozygosity to what would be expected in the absence of population structure [68]. We calculated three values using GenAlEx [69]: (i) *F*_IT_, the reduction in heterozygosity of individuals relative to the entire population due to non-random mating; (ii) *F*_ST_, the reduction in heterozygosity due to population subdivision (indicating reproductive isolation between breeding groups); and (iii) *F*_IS_, the reduction in heterozygosity of individuals relative to their social group, caused by non-random mating within groups.

To investigate further the genetic structure of our study population, we conducted a Bayesian cluster analysis using STRUCTURE v2.3.1. [70]. This program uses a maximum likelihood approach to determine the most likely number of distinct genetic clusters in the sample (*K*) and individual membership to each cluster. We ran six independent runs for *K* = 1−14 using 100 000 Markov chain Monte Carlo (MCMC) iterations after a burn-in of 100 000, assuming independent allele frequencies and admixture. We used the ‘locprior’ model, which uses knowledge of the groups from which individuals originated when assigning individuals to genetic clusters as this model outperforms the standard model when populations are weakly differentiated, therefore generating more accurate estimates of *K*. The most likely value of *K* was evaluated using Ln *P*(*D*), which measures the maximal average value of the posterior probability of the data, and delta *K*, an ad hoc statistic based on the rate of change of the likelihood of *K* [71] (electronic supplementary material, figures S1 and S2). To assess the potential for spatial genetic structure, we conducted a Mantel test on between-group relatedness values and a binary measure of whether groups were neighbours. Neighbours were assigned as groups that shared a territorial boundary and had been witnessed engaging in at least one inter-group interaction during the study period.

#### 2.3.4. Relatedness analysis

We calculated Queller & Goodnight’s [72] pairwise relatedness values between all individuals (excluding those with <7 loci genotyped; number included = 89 individuals) using GenAlEx [69]. To assess whether the relatedness between the dominant breeding pair within each group was lower than if individuals mated with groupmates at random, we conducted a permutation test. For each dominant individual, we randomly sampled all opposite-sex groupmates with known genetic relatedness to the dominant, generating a mean relatedness between these permuted pairs. This process was repeated to generate a distribution of 1000 within-group relatedness measures to compare to the observed breeding pair relatedness to assess significance.

#### 2.3.5. Parentage analysis

We conducted parentage analysis using Cervus 3.0.7 [67]. Cervus uses a likelihood approach to assign the most likely maternity and paternity. We included as potential mothers all females that were in the same group as the pup and were recorded as being pregnant within two months of the pup being born. As potential fathers, we included all males in the same social group as the pup and who were over 2 years old. Cervus establishes parentage confidence using simulations, which were set according to the following parameters (based on our dataset): two candidate mothers and two candidate fathers per offspring, 0.48 and 0.47 of candidate mothers and fathers sampled, respectively, 0.91 loci genotyped, 0.05 loci mistyped, minimum number of genotyped loci = 7, 0.8 of candidate females were related to the mother by 0.31 and 0.8 of candidate fathers were related to the father by 0.27. We determined parentage confidence using delta (the difference in likelihood score between the most likely and second most likely parent pairs for each pup).

As our behavioural observations indicated that the dominant pair were most likely to breed, we restricted downstream analysis of the parentage results to the 35 pups where both dominant potential parents had been genotyped. For all 35 pups, we were able to assign a mother at >80% confidence, with 27 (77%) of these assignments being at >95% confidence. For 31 pups, we also assigned a father at >80% confidence, with 20 pups (57%) being assigned at >95% confidence. As there is the potential for extra-group mating, we also tried repeating the parentage analysis including all males in the population as potential fathers (rather than restricting paternity analysis to the social group). However, this made very little difference to the parentage results; the identity of the assigned father did not change for any pup, but fathers were assigned at lower confidence due to comparing a greater number of potential parent pairs.

## 3. Results

### 3.1. Life-history summary

**Figure 1.**
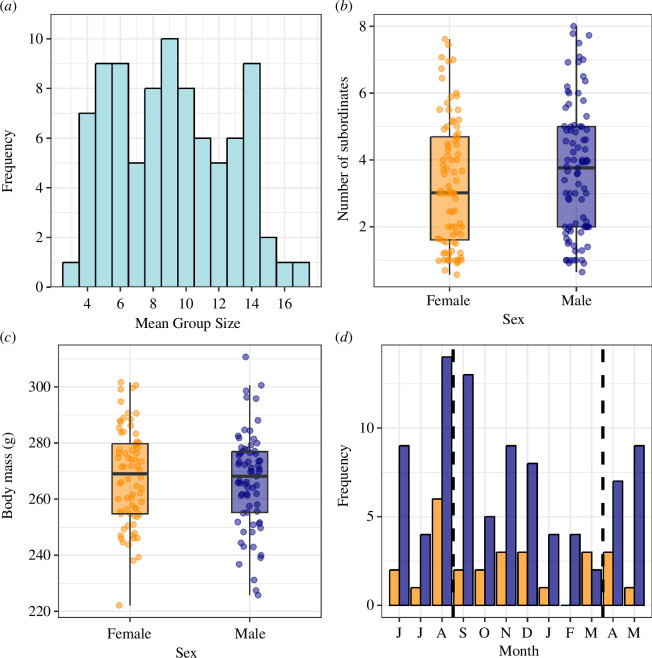
(*a*) Mean weighted group size per group-season, (*b*) mean number of subordinates per sex per group per group-season and (*c*) mean adult body mass per sex in the breeding season. (*a,b*) *N*_Breeding Seasons_ = 12, *N*_Groups_ = 13, *N*_Group-seasons_ = 87; (*c*) *N*_Breeding Seasons_ = 12, *N*_Groups_ = 12, *N*_Group-seasons_ = 72, *N*_Weights_ = 29 732, *N*_Means_ = 141. Boxplots denote median plus quartiles, with arms representing median ± 1.5× interquartile range. (*d*) Number of group-switching events per month, split by sex (*n* = 112). Dashed black lines denote mean start and end dates of the breeding season across the study period; orange = female, blue = male.

Across the 12 breeding seasons monitored, groups contained on average 8.2 ± 0.6 (mean ± SE) individuals born before the onset of that breeding season (group-season weighted-mean range: 3−17.2; absolute range: 3−21; 73 group-seasons; figure 1*a*). Groups contained 3.2 ± 0.3 male (season range: 0.6−10.1; absolute range: 0−12) and 2.9 ± 0.3 female (season range: 0−9.5; absolute range: 0−10) subordinates. There was no significant difference in the number of subordinate helpers of each sex in each group (LRT: *χ* ^2^_1_ = 2.07, *p* = 0.15; table 1*a*). Once periods when females were known to be pregnant were excluded, there was no significant difference between the body mass of males (266.4 ± 0.3 g) and females (268.0 ± 0.2 g) in breeding seasons (*χ* ^2^ = 0.38, *p* = 0.54; table 1*b* and figure 1*b*).

**Table 1.** Outputs from LMMs assessing sex differences in (*a*) the number of subordinates per breeding season (*N*_Means_ = 174, *N*_Groups_ = 13, *N*_Seasons_ = 12) and (*b*) mean body mass per breeding season (*N*_Means_ = 141, *N*_Groups_ = 12, *N*_Seasons_ = 10). For the fixed term sex, female was the reference level. (*c*) Output from a generalized LMM (binomial error structure and logit link function) assessing if there were sex differences in whether a group-switch event involved a coalition (*N*_Switches_ = 112). (*a*–*c*) Random term variance (s.d.) italicized.

factor	estimate ± SE	*χ^2^*	d.f.	*p*
(*a*) number of subordinates				
intercept	3.00 ± 0.34			
sex_Male_	0.37 ± 0.26	2.07	1	0.15
*group*	*0.73 (0.85)*			
*season*	*0.25 (0.50)*			
(*b*) body mass (g)				
intercept	268.09 ± 3.20			
sex_Male_	−1.58 ± 2.56	0.38	1	0.54
*group*	*6.30 (2.51)*			
*season*	*62.65 (7.92)*			
(*c*) group switch in coalition? (Y/N)				
intercept	−12.02 ± 3.19			
sex_Male_	0.72 ± 3.07	0.06	1	0.80
*group-switch event*	2555 (50.55)			

Over the study period, 112 individuals were confirmed to have moved between groups, with a male-biased ratio of 3 : 1 (86 males and 26 females). The majority of the 74 group-switch events were by a single individual (*n* = 54 events), but over half of the individuals observed moving between groups were part of a coalition (*N*_Events_ = 20, *N*_Individuals_ = 58, coalition size range = 2−7 individuals). Of these 20 multi-individual group switches, the majority (*n* = 15) were all-male coalitions, two were all-female and three were mixed-sex. There was no significant difference between the sexes in the likelihood of dispersing in a coalition (51/86 males and 10/26 females) versus alone (LRT: *χ* ^2^_1_ = 0.06, *p* = 0.80; table 1*c*). Group switches occurred year-round but appear to have been most prevalent around the beginning of the breeding season (figure 1*c*), a pattern that appears consistent in both sexes. Of group switches where we were actively monitoring the group immigrated into, 10 immigrants (three females and seven males) immediately occupied a breeding position on switching, whereas 77 (19 females and 58 males) entered the group as a subordinates. Six of these immediate breeding occupancies were the result of takeovers, where the immigrating individual ousted a same-sex dominant, while the other four were instances of an immigrant immediately filling a breeding vacancy. Of all group-switch events recorded, only one active eviction of a subordinate of each sex was witnessed. When comparing group-switch events where both the group emigrated from and the group immigrated into were part of the study population, there was weak evidence that switching reduced the number of same-sex competitors. Each group switch reduced the focal individual’s number of same-sex groupmates by 0.6 individuals, but not significantly (one-sample *t*‐test: *t* = −1.90, *p* = 0.07), with 18/38 switches decreasing and 14/38 increasing the number of same-sex subordinates in the group (range = 5 fewer to 3 more same-sex groupmates). At the start of each breeding season, a mean of 18.5% of individuals were known immigrants to the group, with the remainder known to be natal to the group (data from the 181 individuals of known origin). In line with the male-biased dispersal seen, group origin was sex-biased, with only 6% of female individuals coming from a different group, compared to 29% of males.

**Figure 2.**
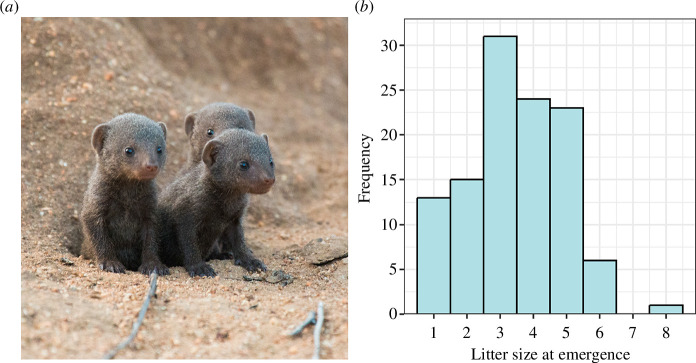
(*a*) A litter of dwarf mongoose pups a week after emerging from the burrow (5 weeks old). (*b*) Histogram of litter sizes at emergence. *N*_Litters_ = 113, *N*_Groups_ = 12, *N*_Seasons_ = 11.

Per group-season, we observed 2.0 ± 0.6 (mean ± SE, range: 1−6) different males mating with an adult female and 1.7 ± 0.5 females (range: 1−6) mating with an adult male. This led to 1.8 ± 0.1 females becoming pregnant per group-season (range: 1−5, all data from 12 groups, 78 group-seasons). Of the 176 pregnancy events witnessed, a subordinate was recorded as pregnant during 72. Of these 176 events, there were 69 instances (39.2%) where no pups were observed emerging from the burrow. This could be due to a combination of abortion, infanticide and predation, although the cause of mortality at this life-stage is largely unknown. Including pregnancies that were not directly observed (due to groups not being found or temporarily moving outside of the study area), 176 litters were produced in total, with 2.2 ± 0.01 per group-season. In all but one season, each group produced between one and three litters, but in one group-season, a subordinate female gave birth asynchronously with the dominant female, resulting in a fourth distinct litter that emerged from the burrow. For those litters that did emerge from the burrow, litter size at emergence was 3.5 ± 0.1 (range: 1−8, *n* = 113 litters; figure 2). Of pups that emerged, 39% survived to 1 year (211/538). However, seven individuals were known to emigrate out of the study population before reaching 1 year old (age range 274−323 days old), so survival to 1 year may be slightly higher.

### 3.2. Genetic analysis

**Figure 3.**
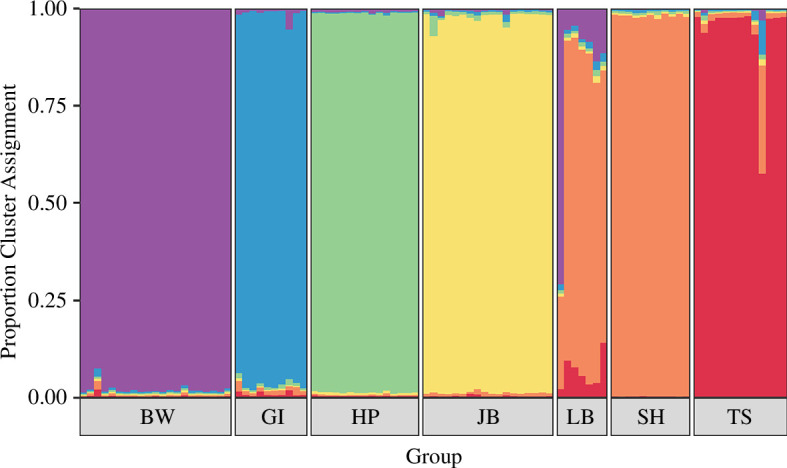
Cluster membership coefficients derived from the program STRUCTURE at *K* = 6. Each vertical bar represents an individual, and the coloured segments represent the posterior probability of membership to each of the six clusters.

Social groups were significantly genetically distinct (*F*_ST_ = 0.192, *p *< 0.001). Group genetic relatedness (*F*_ST_) was unrelated to whether groups were territorial neighbours or not (Mantel test, *p* = 1). STRUCTURE analysis identified six distinct genetic clusters within the sampled population of seven groups, with Ln values and Δ*K* both peaking at 6 (electronic supplementary material, figures S1 and S2). This suggests that one pair of groups (LB and SH) had shared genetic ancestry that predates their inclusion in the study system (highlighted in orange in figure 3). This clustering also identified the one individual known to have switched groups within the genotyped sample: an individual that moved from its natal BW group into the LB group, giving it a majority assignment in the cluster that best represents BW (figure 3).

**Figure 4.**
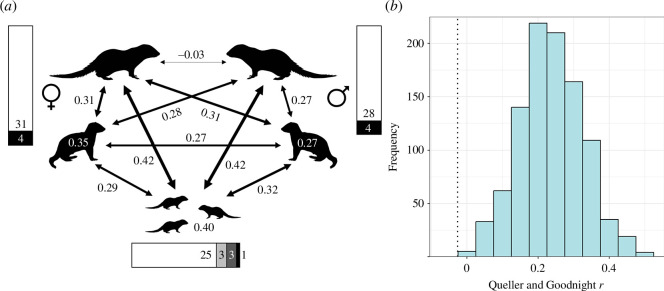
(*a*) Within-group relatedness between each distinct class of individual. The dominant pair are depicted at the top, above subordinate adults and then pups. Arrows denote pairwise relatedness, with line thickness proportional to relatedness. Numbers within silhouettes denote within-class relatedness (e.g. 0.35 between subordinate females). Side bars denote the number of offspring assigned to dominant (white) and subordinate (black) parents for each sex, with the bottom bar highlighting the parental makeup of each pup where both parents were assigned: white = both dominant; light grey = dominant male, subordinate female; dark grey = dominant female, subordinate male; black = both subordinate. Mongoose silhouettes by Rebecca Groom and Margot Michaud reproduced under creative commons CC BY-SA 3.0 DEED licence https://creativecommons.org/licenses/by-sa/3.0/. (*b*) Histogram of permuted breeder relatedness values from 1000 randomizations. Observed dominant breeder relatedness represented by dashed line.

The mean relatedness between members of a dominant breeding pair was lower than between any other combination of individual classes within a social group. Dominant pairs had an average relatedness of −0.03, equivalent to that of the population average (0), while all other potential within-group mates had a significantly higher average relatedness of 0.28 for dominant males and 0.31 for dominant females (permutation test: *p* < 0.001, *n* = 7 groups; figure 4). The lack of a genetic relationship between dominant breeders is further evidenced by the mongooses in our study being significantly more heterozygous than expected if reproduction was occurring randomly with respect to relatedness within social groups (*F*_IS_ = −0.252, *p* < 0.001). In addition, within-group relatedness among subordinate females (*R* = 0.35) was, on average, higher than among subordinate males (*R* = 0.27), in line with what would be expected given the observed male-biased patterns of dispersal.

Overall, the reproductive skew was high. Nearly 90% of genotyped pups were those of the dominant female (31/35), with just four offspring produced by within-group subordinate females (figure 4*a*). Pups of subordinate females were present in three litters, with one each in two litters and two in one litter; in all three cases, the dominant female also produced pups in the same litter (1/5, 1/5, 2/5 pups produced by the subordinate mother out of total litter size). Similarly, we were only able to rule out the dominant male as a father for four of the 35 pups; three pups were assigned a mother with high confidence, but confidence in the father was low. However, the dominant male was not excluded as a candidate in any of these cases and is therefore the most likely father. Three of the pups confirmed to be fathered by a subordinate were fathered by the same subordinate male in the same litter. This breeding attempt was also one in which a subordinate female produced one pup, with this litter, therefore, containing offspring with four different parents in three combinations (figure 4*a*). This contrasts with the majority of cases, where all pups within a litter shared the same parents (25/32 pups). The mean relatedness for the four parent combinations that contained at least one subordinate was 0.05 (range −0.19 to 0.18), while the individuals that produced the only subordinate–subordinate offspring had a relatedness of 0.15.

Reproductive skew is also apparent through higher average relatedness values between the dominant pair and pups (0.42 for both males and females; figure 4*a*) than between subordinate adults and pups (0.29 and 0.32 for males and females, respectively; figure 4*a*). Such reproductive skew also yielded a high mean within-pup relatedness of 0.40 and an even higher mean within-litter relatedness of 0.47, with one litter containing a pair of monozygotic twins. This genetic reproductive skew is also higher than would be expected given behavioural observations alone. If each observed pregnancy were to result in the same number of pups, we would expect only 55% of offspring to be those of the dominant female (as opposed to the observed 88.6%). Similarly, if every male who was observed mating with a female had an equal chance of fathering pups, we would expect subordinate males to sire 50% of offspring (as opposed to the observed 12.5%).

## 4. Discussion

Combining long-term life-history observations with genetic sampling, we describe key aspects of social and breeding structure in a South African dwarf mongoose population. Groups contained equal numbers of male and female subordinate helpers, who had similar body mass. Switches between groups were male-biased and concentrated around the onset of the breeding season. However, most group switches did not result in immediate access to a dominant breeding position. Despite the occurrence of matings by, and pregnancies among, subordinate individuals, dominants produced almost 90% of offspring and thus there was high reproductive skew. Groups produced an average of eight pups per season, across multiple litters, fewer than half of whom survived to 1 year of age. The population exhibited genetic diversity indicative of inbreeding avoidance, backed up by the finding that dominant breeding pairs were unrelated, while subordinates were generally highly related to their groupmates. Taken together, our work shows the many avenues to both indirect and direct reproductive fitness available to dwarf mongooses and the population-level genetic patterns that these produce.

The dispersal patterns in our study population showed variability and nuance in who disperses, when and with whom. Whilst the observed male-biased dispersal fits that described generally for mammalian systems [73], females also regularly switched groups at rates in line with the previous study of the species in Tanzania [52,74]. This switching in both sexes is likely driven by decreases in inclusive fitness as helpers become less likely to be related to breeding dominants as they age [75]. The level of female immigration into new existing groups seen in dwarf mongooses therefore contrasts with that seen in closely related meerkats, where females are more often evicted than proactively emigrate [76,77], and banded mongooses, where females form new groups with coalitions of dispersing males [36]. Similar to other mongoose species, more than half of individuals moved as part of a coalition. Leaving together may have increased the chances of successful dispersal in a harsh environment (as in pied babblers [78]) through, for example, cooperative anti-predator defence, as well as providing cooperative partners in a new group, analogous to the situation in long-tailed tits [79]. The increase in the number of individuals switching groups around the onset of the breeding season suggests a link between group-switch timing and reproductive potential. Evictions of reproductive rivals are common in similar species such as meerkats [80], but the rarity of observed evictions in our study population make this unlikely to be the sole cause of this pattern in group-switch timing. Despite this timing, the majority of dwarf mongoose immigrants enter the group as subordinates, as is the case in meerkats [76]. Dwarf mongoose immigrants take roughly six months to become fully integrated and treated as a resident group member due to the costs of dispersal, performing less sentinel behaviour than residents [81]. This peak in timing of group switches therefore suggests that full integration into the group may not be necessary to access potential reproductive opportunities.

Genetic analyses revealed a high level of reproductive skew in our population, substantially above that which would be expected from observations of matings and pregnancies alone. Previous analysis suggested we should not expect a direct relationship between mating patterns and reproduction due to some mated individuals having low fertility [54]. Furthermore, the discrepancy between observed pregnancy rates and ultimate contributions to offspring points to breeding suppression above the behavioural and endocrine mechanisms that prevent subordinate pregnancy previously described in the species [54]. Although births happen almost exclusively below ground and therefore unobserved, infanticide has occasionally been witnessed in our population and could help explain the observed lack of subordinate reproductive success. Pregnant subordinates appear to attempt to synchronize their reproduction with the dominant female, a strategy that has been demonstrated to increase subordinate reproductive success in banded mongooses [82,83]. The subordinate reproduction we observed is not necessarily a failing of reproductive suppression by the dominant individuals, but an adaptive response to current circumstances. Theoretical modelling predicts that payoffs for dispersal increase relative to those accrued by helping in the natal group as subordinates age [84], so dominants may allow some subordinate reproduction to retain valuable experienced helpers. This is in line with observations in Tanzania where subordinates reproduced at comparable rates [33].

We found evidence for genetic structure between groups within our population, as well as genetic diversity indicative of inbreeding avoidance. The general avoidance of breeding with close relatives in the South African population is apparently contrary to findings in Tanzania [57], but our updated genetic methodology likely provides a more accurate picture. Despite South African groups forming genetic clusters with high within-group relatedness, dominant breeding individuals were less related than under random mating scenarios and often the least related dyad within their group. The high degree of heterozygosity, coupled with low relatedness within dominant breeding pairs, highlights the existence of a mechanism for dwarf mongooses to largely avoid mating with close relatives. This mechanism need not be sophisticated; meerkats, for example, appear only to avoid inbreeding with relatives they are familiar with, suggesting that a proxy for relatedness based on group membership is used [85–87]. This strategy of kin recognition will likely be complicated by synchrony in births [88], such as when subordinates produce pups that are raised alongside those of dominants, but the high levels of reproductive skew may enable such simple rules to be sufficient. Finally, the lack of differential relatedness between neighbours and non-neighbours suggests that individuals are dispersing more widely than to the nearest groups. This is similar to dispersal patterns of pied babblers, where individuals disperse twice as far when leaving a natal versus a non-natal group, as travelling larger distances should increase the chances of finding unrelated breeding partners [89]. The evidence for non-neighbour dispersal also increases the likelihood that our observational data underestimate the rates of emigration as individuals often go missing from their group, but we cannot know whether they have been depredated or dispersed outside of the study area.

Taken together, our data demonstrate multiple potential avenues by which individuals can obtain reproductive success. The evidence for some, limited, subordinate reproduction highlights how non-dominants can occasionally access direct reproductive success without the need to emigrate. However, subordinates were, on average, closer related to pups born in their group than they would be to their direct descendent grandchildren (*r* > 0.25), highlighting the potential inclusive fitness benefits of helping to raise the litters of a closely related dominant pair [84]. Benefits of remaining and helping in a natal group are further highlighted by the costs of dispersal in such a predator-rich environment [90] and the relatively high levels of pup mortality observed, rendering dispersal risky and help valuable. While it is possible that switching groups can provide individuals with fewer potential competitors and less related potential partners (as in Rood [56]), the majority of individuals still had to wait for direct reproductive opportunities, as they entered their new group as a subordinate. Switching groups therefore trades off a potential increase in the chance of direct reproduction against providing help for less related individuals in the new group. Finally, we found no evidence that out-group males are regularly contributing to reproduction, contrasting with banded mongooses where out-group males contribute up to a quarter of paternity [91].

Our findings, coupled with those from Tanzania, show broad similarities between the social and genetic structure of dwarf mongoose populations at opposite ends of their range. Reproductive skew, rates of subordinate reproduction and immigration dynamics all appear broadly consistent despite the geographical separation of approximately 2500 km. However, we did find differences in genetic diversity between the populations, such that in our South African population inbreeding was avoided and relatedness between breeders was less than between breeders and other groupmates. This could be in part due to the lower density of dwarf mongooses living in Tanzania: despite the Serengeti receiving more annual rainfall than our study site and exhibiting greater climatic stability [92], Tanzanian mongoose groups had home ranges with a mean size of 34.6 ha (those in open habitat can be up to 160 ha), which is 10 ha larger than in our study population [52] (DMRP, unpublished data). Lower population density could result in fewer unrelated potential breeding partners and thus an increased inbreeding risk. The larger home ranges in the Tanzanian population may also increase the dispersal distance required to find unrelated individuals, potentially further suppressing gene flow and increasing inbreeding risk, although this pattern has not been observed in banded mongooses [93]. The higher climatic stability seen in Tanzania compared to South Africa may be a contributing factor to the slightly higher offspring survival rates seen. As groups were comparable in number of subordinate helpers, the increased rainfall and stable annual temperatures may create a less harsh natal environment, resulting in higher pup survival.

In summary, our results demonstrate how dwarf mongoose dominants monopolize reproduction, while subordinates are occasionally able to gain direct reproductive benefits. This results in strong genetic structuring and high relatedness within groups, factors likely to promote helping behaviour over costly dispersal or personal reproduction. This study also shows that there is overall consistency in dwarf mongoose genetic and social structure at opposite ends of their geographic range despite contrasts in ecology. Future work should investigate whether these results are matched in populations living at ecological extremes (such as those in arid regions of Somalia and Namibia), and how stable socio-genetic structures are across generations. Our work highlights mismatches between behavioural observations of mating and pregnancy relative to reproductive success, making obvious the benefits of integrating genetic data with long-term behavioural and life-history observations to produce a clearer picture of the breeding ecology and population structure of a species. More generally, our study adds to the incremental process of furthering understanding of the evolution and maintenance of cooperative breeding.

## Data Availability

All data required to generate statistical and genetic outputs are included as electronic supplementary material [94].
